# It's Time to Get Practical: A Call for Shorter Measures in the Family Planning Community

**DOI:** 10.1111/sifp.70056

**Published:** 2026-05-08

**Authors:** Lotus McDougal

## Abstract

While there is widespread agreement that measurement is an essential component of successful family planning programming, policy‐making, and research, current measures are still catching up to the field's shift to more person‐centered and agency‐focused goals. Concurrently, the global reproductive health and data ecosystems have been profoundly interrupted, and resources are becoming increasingly scarce. This is thus a critical time to reexamine our priorities regarding what and how we measure across different aspects of family planning. In the last decade, new family planning measures had an average of 14 items per measure. This length is impractical for inclusion in most large‐scale surveys and unfeasible in many modalities of data collection. In order to meet the very difficult moment in which the family planning community now finds itself, we must embrace pragmatism and promote the development, testing, and use of reliable, valid, and short (five or fewer item) measures. We have a chance to be both responsive and adaptive, and it is timely and necessary that we do so as a family planning measurement community.

## THE NEED FOR MEASUREMENT

Measuring progress is an essential component of family planning policy and programs. Measurement allows us to track progress and recognize trends, and informs what we understand about individual, programmatic, policy, and structural needs within family planning. The importance of measurement is clear: we cannot effectively design, monitor, and evaluate programs and policies, nor can we advocate—for funds, for services, for programs, for global targets, for rights—with what we cannot quantify and track. And we cannot quantify and track what we cannot measure.

The family planning community has been in the midst of a measurement landscape evolution for several years now. This has been precipitated in part by an ongoing transition towards the prioritization of person‐centered programming (Bhan and Raj 2021; Holt et al. 2017). As we shift from goals that are behaviorally based, such as contraceptive prevalence, to those that center reproductive choice and agency, such as the achievement of self‐determined goals for contraceptive use (or nonuse), our measures must also shift (Speizer, Sully, Hashem, et al. 2025; Rothschild et al. 2025). This transition is complex, as—in addition to contraceptive behaviors—it commensurately necessitates the measurement of individual needs, preferences, and desires, as well as individual goals and satisfaction. These components sit alongside the myriad of interpersonal, community, social, and structural factors that also foundationally influence family planning.

Perhaps unsurprisingly, within this complex context, the family planning community has struggled to identify and align on the core metrics that can be used over the next several decades to understand whether and how women, men, and families are achieving their reproductive goals (Speizer, Bremner, and Farid 2022; Fabic 2022; Speizer, Sully, Binstock, et al. 2025). The shift from behavior‐based measures to agency‐focused and person‐centered measures has underscored key gaps in our measurement repositories. We lack the necessary tools to capture many of these new foci (Speizer, Sully, Hashem, et al. 2025; Rothschild et al. 2025). While work to fill these gaps is ongoing, and indeed has been catalyzed in recent years (Holt et al. 2023; Bullington et al. 2025; Senderowicz 2020; Rothschild, Brown, and Drake 2021; Vincent et al. 2025), we must also acknowledge that many of our existing measures are imperfect, and particularly, impractical.

## THE ARCHITECTURE OF MEASUREMENT

The science of measuring psychosocial phenomena comes largely from the field of psychology, where scientists developed methods to capture complex latent constructs through a series of questions that, when considered as a whole, represent that construct (Jones and Thissen 2006; DeVellis 2016). These methods broadly involve the development and refinement of a pool of questions that measure different aspects of the construct of interest, which are cognitively assessed, refined, and subsequently enumerated in a sample of people that represent the population in which one is eventually interested in measuring the construct in question (DeVellis 2016; Boateng et al. 2018). These scales are then assessed for key psychometric properties such as reliability and validity, to test whether item sets produce consistent results, and measure what they are meant to. The final items are generally aggregated into a composite score (and sub‐scores, if the construct of interest includes sub‐constructs).

This process has been developed and refined over decades and is well‐established. It has enabled meaningful advancements in our ability to empirically measure complex phenomena that cannot be directly observed. But this approach is predisposed to favor longer scales, both conceptually and mathematically. Measuring unobservable phenomena often comprises a broad item pool to ensure that different aspects of the conceptual framing of a given phenomenon are represented. While item reduction techniques help with the transition from a larger item pool to a smaller proposed scale, key statistical methodologies used to assess scale performance are tied to measure length. For example, Cronbach's alpha, a statistic commonly used to measure the internal reliability of a given measure, tends to be higher and more precise as the number of assessed items increases, provided those items are reasonably good fits for the measure in question and are positively intercorrelated (DeVellis 2016; Cortina 1993).

What longer measures gain in precision and nuance, they generally sacrifice in terms of applied utility. Measures that are 10, 15, 20, or more items may be able to advance our understanding of the manifestations of complex phenomena—provided their questions are clear, and have demonstrated reliability and validity in their focal populations—but we cannot overestimate the challenges in implementing these measures at scale.

## THE ANATOMY OF MEASUREMENT

**FIGURE 1:**
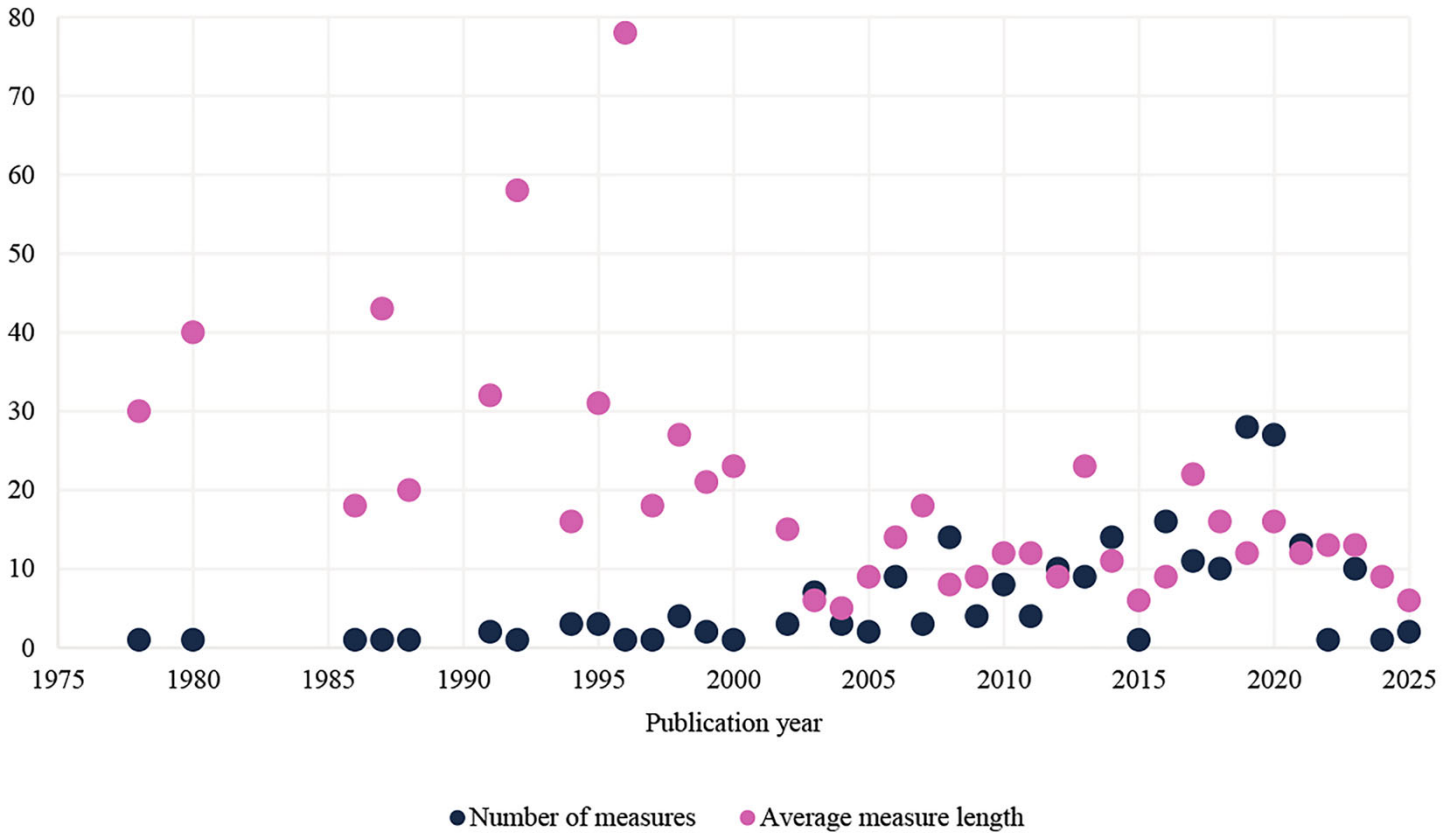
Summary of sexual and reproductive health and family planning measures included in the EMERGE online measure repository

The development of family planning measures that are shorter in length has been a trend for several decades. A February 2026 review of the EMERGE website, an online repository of gender equity and empowerment measures, identified 233 measures focused on sexual and reproductive health and family planning published over the past 50 years (Center on Gender Equity and Health 2026). While the number of new measures has generally increased in this period, the average number of items in each of those measures has decreased, from 35 items/measure in 1975–1985, to 29 items/measure in 1986–1995, to 16 items/measure in 1996–2005, and only 12 items/measure in 2006–2015 (Figure 1). However, there has actually been a slight overall increase in the 2016–2025 period, to 14 items/measure, though 2024 and 2025 again indicated a decrease in average measure length.^1^

These trends highlight several important findings. First, we are publishing more family planning measures over time, likely reflecting both the growing priority of the importance of measuring family planning and an increasing breadth of family planning‐related constructs people believe are meaningful to quantify. Second, new measures are generally getting shorter—we have reduced the average number of items per sexual and reproductive health and family planning measure by more than half in the last 50 years. However, the increase in measure length in the past decade is concerning, suggesting that this trend may be plateauing, or even reversing. The decline in measure length since 2024 is hopeful, but requires ongoing monitoring to determine if the decline will persist.

As a field, our existing measures are thus generally in the 10–15 item range. For many applications, these measures are prohibitively long and demand an untenable amount of “real estate” in a given survey, particularly if that survey is designed to address topics beyond family planning. This includes cross‐national surveys such as the Demographic and Health Surveys, which inform global measurement and progress agendas like the Sustainable Development Goals (SDGs), as well as surveys that will be needed in the post‐SDG era (Department of Economic and Social Affairs 2026). Longer measures are problematic in surveys that aim for brevity to reduce costs or respondent burden/fatigue, or that are limited in the number of questions that can be feasibly asked (e.g., SMS or digital surveys) (Dabalen et al. 2016; Glazerman et al. 2020). They also carry a greater risk that one or more items in that measure will not perform well in a particular context (D'Urso et al. 2022).

Longer, more precise, and nuanced measures are important—they offer insights into the construct of interest that cannot be obtained through shorter, more parsimonious measures. Longer measures may be appropriate, necessary, and beneficial for specific circumstances. These could include program evaluations or randomized controlled trials in which the measure represents a key construct of interest relevant to the program in question, and where understanding varying performance within construct sub‐domains could differentially influence program modifications or assessments of success. This specificity, however, comes at a price—it costs resources, in terms of money, time, survey length, and often survey complexity, to administer longer measures.

## THE PRACTICALITY OF MEASUREMENT

As a field, we must recognize the resource scarcity in which we are living, and come together to ensure that the ever‐increasingly limited resources available for family planning are used effectively and efficiently. I urge us to collectively endorse our prioritization of practical, short, clear, simple measures that are no longer than five items in length, and ideally shorter than three items. While single‐item measures may be unfeasible and overly simplistic for more complex constructs, working within this range offers some flexibility balanced with a heavy emphasis on pragmatism. This brevity reflects a goal of developing measures suitable for use as global indicators and for inclusion in large‐scale, multi‐topic surveys. It is similarly important for addressing the impacts of longer surveys on response and completion rates, particularly for digital modalities (Galesic and Bosnjak 2009; Edwards et al. 2023; Dabalen et al. 2016; Glazerman et al. 2020).

Some of this work is well underway. For example, there are currently several proposed measures that assess the concordance between contraceptive desire/preference and contraceptive behavior (Holt et al. 2025; Vincent et al. 2025; Bullington et al. 2025). These measures essentially ask versions of “do you want to be using contraception” and “are you using contraception” and assess alignment between the two. Particularly given that most surveys in which questions like this would be enumerated are already asking about contraceptive behavior, the additional question burden is minimal. In return, one is able to calculate a proxy of contraceptive agency by assessing a self‐determined goal and whether or not that goal has been achieved. While there is not yet consensus on a final item set for this family of measures, the fact that these short, practical, and person‐centered measures are being convergently developed shows great promise.

The goal of reducing family planning measure length cannot be solely operationalized at the measurement level. Measures are generally driven by and reflective of key concepts, constructs, and outcomes that are important to different stakeholders in the family planning field, including policy‐makers, practitioners, implementers, funders, and ultimately, women and men. This breadth, alongside the lack of an agreed upon “north star” towards which we are all working, is one of the main struggles faced by family planning. Different stakeholders have different priorities, goals, and mandates, which inform their measurement frameworks and the measures they develop, adapt, implement, and track. Aligning, streamlining, and simplifying these measurement frameworks would substantially and meaningfully facilitate the streamlining of the measures used to monitor their progress. This alignment is perhaps a loftier goal towards which to work, but it carries manifold benefits, and some organizations are already active in this space (International Union for the Scientific Study of Population 2026; United Nations Population Fund 2026; FP2030 2023).

In the face of interrupted global data systems and funding, from a measurement perspective, this call for practicality means adhering to the adage of not letting perfect be the enemy of good. Shorter measures tend to be simpler to understand, less expensive to collect, and more feasible to incorporate in a range of data collection modalities and contexts. To be sure, this means that there will be compromises in measurement complexity and nuance. We will not be able to measure every subdomain or conceptual underpinning of a complex construct. We will not be able to capture all of the distinct aspects of a particular concept. And it is the responsibility of the family planning measurement community to ensure that these shorter measures do in fact measure what they purport to, are reliable and valid, and serve their intended goals. But by prioritizing development, widespread testing, and use of measures that are five items or fewer, we gain the potential to meet the needs of the field to have measures that are more feasible to implement, that are more responsive to the needs of a data‐scarce environment with fewer household surveys, and that ultimately are more practical.

## CONFLICTS OF INTEREST STATEMENT

The author declares no conflict of interest.

## Data Availability

Data from the EMERGE database is publicly available at https://emerge.ucsd.edu.

## Footnotes

1 The EMERGE repository has not been regularly updated with new measures since September 2025 due to funding limitations; therefore, 2025 estimates may not be reflective of all new measures published in that year.
